# Bis(dicyanamido-κ*N*)tetra­kis­(pyridazine-κ*N*)nickel(II)

**DOI:** 10.1107/S1600536812018363

**Published:** 2012-04-28

**Authors:** Susanne Wöhlert, Mario Wriedt, Inke Jess, Christian Näther

**Affiliations:** a Institut für Anorganische Chemie, Christian-Albrechts-Universität Kiel, Max-Eyth-Strasse 2, 24118 Kiel, Germany; b Departement of Chemistry, Texas A&M University, College Station, Texas 77843, USA

## Abstract

Reaction of nickel(II) chloride with sodium dicyanamide and pyridazine leads to single crystals of the title compound, [Ni{N(CN)_2_}_2_(C_4_H_4_N_2_)_4_], in which the Ni^II^ cation is octa­hedrally coordinated by two dicyanamide anions and four pyridazine ligands into a discrete complex that is located on a center of inversion.

## Related literature

For the synthesis, structures and properties of dicyanamide coordination compounds, see: Wriedt & Näther (2011 ▶).
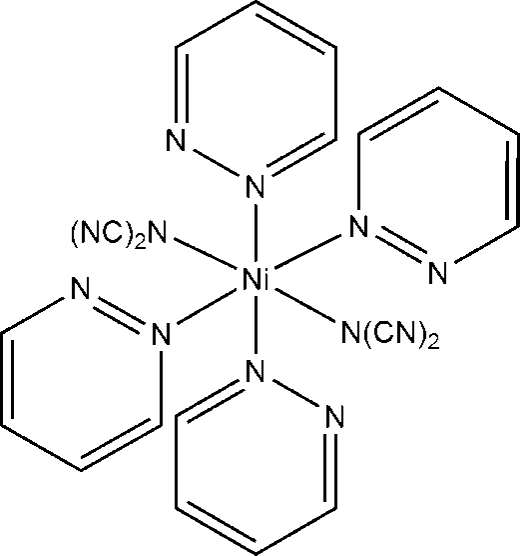


## Experimental

### 

#### Crystal data

[Ni(C_2_N_3_)_2_(C_4_H_4_N_2_)_4_]
*M* *_r_* = 511.18
Triclinic, 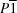

*a* = 8.1796 (12) Å
*b* = 8.4125 (12) Å
*c* = 8.9643 (11) Å
α = 81.364 (16)°
β = 66.027 (15)°
γ = 84.879 (17)°
*V* = 556.97 (13) Å^3^
*Z* = 1
Mo *K*α radiation
μ = 0.91 mm^−1^
*T* = 170 K
0.10 × 0.08 × 0.06 mm

#### Data collection

Stoe IPDS-1 diffractometer
Absorption correction: numerical (*X-SHAPE* and *X-RED32*; Stoe & Cie, 2008 ▶) *T*_min_ = 0.783, *T*_max_ = 0.927
4159 measured reflections
2142 independent reflections
1582 reflections with *I* > 2σ(*I*)
*R*_int_ = 0.068

#### Refinement

*R*[*F* ^2^ > 2σ(*F* ^2^)] = 0.048
*wR*(*F* ^2^) = 0.097
*S* = 1.01
2142 reflections
161 parameters
H-atom parameters constrained
Δρ_max_ = 0.51 e Å^−3^
Δρ_min_ = −0.52 e Å^−3^

### 

Data collection: *X-AREA* (Stoe & Cie, 2008 ▶); cell refinement: *X-AREA*; data reduction: *X-AREA*; program(s) used to solve structure: *SHELXS97* (Sheldrick, 2008 ▶); program(s) used to refine structure: *SHELXL97* (Sheldrick, 2008 ▶); molecular graphics: *XP* in *SHELXTL* (Sheldrick, 2008 ▶) and *DIAMOND* (Brandenburg, 2011 ▶).; software used to prepare material for publication: *XCIF* in *SHELXTL*.

## Supplementary Material

Crystal structure: contains datablock(s) I, global. DOI: 10.1107/S1600536812018363/bt5900sup1.cif

Structure factors: contains datablock(s) I. DOI: 10.1107/S1600536812018363/bt5900Isup2.hkl

Additional supplementary materials:  crystallographic information; 3D view; checkCIF report

## Figures and Tables

**Fig. 1.**
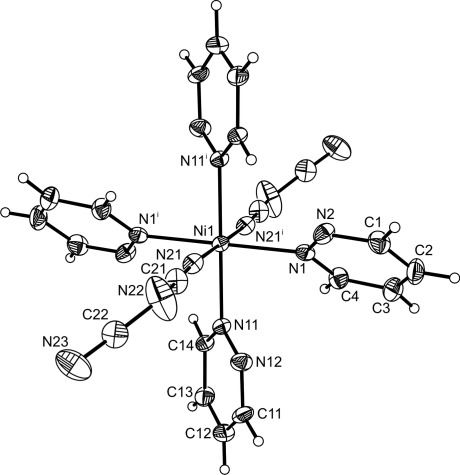
: Crystal structure of the title compund with labelling and displacement ellipsoids drawn at the 50% probability level. Symmetry code: i = -x, -y + 1, -z + 1.

## supplementary crystallographic information

### Comment

Recently we have reported on the synthesis and characterization of paramagnetic transition metal complexes with dicyanamide as anion (Wriedt & Näther, 2011). As a part of our ongoing study in this field the crystal structure of the title compound was determined. The asymmetric unit of the title compound consits of one nickel(II) cation which is located on a center of inversion as well as one dicyanamide anion and two pyridazine ligands both in general position (Fig. 1). In the crystal structure discrete complexes are formed, in which each nickel(II) cation is coordinated by two terminal coordinated dicyanamide anions and four pyridazine ligands in a slightly distorted octahedral geometry. The Ni—N distances are in the range of 2.058 (3) Å to 2.147 (3) Å with the longer distances to the pyridazine ligands. The shortest intermolecular Ni···Ni distance amounts to 8.1796 Å.

### Experimental

Nickel(II) chloride hexahydrate (NiCl_2_x6H_2_O), sodium dicyanamide (NaN(CN)_2_) and pyridazine were obtained from Alfa Aesar. All chemicals were used without further purification. 0.125 mmol (29.7 mg) NiCl_2_x6H_2_O, 0.25 mmol (22.3 mg) NaN(CN)_2_ were reacted in 1.5 ml pyridazine. Green single crystals of the title compound were obtained after one week.

### Refinement

All H atoms were located in difference map but were positioned with idealized geometry and were refined isotropically with *U*_eq_(H) = 1.2 *U*_eq_(C) of the parent atom using a riding model with C—H = 0.95 Å.

### Crystal data

**Table:**

[Ni(C_2_N_3_)_2_(C_4_H_4_N_2_)_4_]	*Z* = 1
*M**_r_* = 511.18	*F*(000) = 262
Triclinic, *P*1	*D*_x_ = 1.524 Mg m^−^^3^
Hall symbol: -P 1	Mo *K*α radiation, λ = 0.71073 Å
*a* = 8.1796 (12) Å	Cell parameters from 4159 reflections
*b* = 8.4125 (12) Å	θ = 2.5–26.0°
*c* = 8.9643 (11) Å	µ = 0.91 mm^−^^1^
α = 81.364 (16)°	*T* = 170 K
β = 66.027 (15)°	Block, green
γ = 84.879 (17)°	0.10 × 0.08 × 0.06 mm
*V* = 556.97 (13) Å^3^	

### Data collection

**Table:**

Stoe IPDS-1 diffractometer	2142 independent reflections
Radiation source: fine-focus sealed tube	1582 reflections with *I* > 2σ(*I*)
Graphite monochromator	*R*_int_ = 0.068
phi scan	θ_max_ = 26.0°, θ_min_ = 2.5°
Absorption correction: numerical (*X-SHAPE* and *X-RED32*; Stoe & Cie, 2008)	*h* = −10→10
*T*_min_ = 0.783, *T*_max_ = 0.927	*k* = −10→10
4159 measured reflections	*l* = −11→11

### Refinement

**Table:**

Refinement on *F*^2^	Secondary atom site location: difference Fourier map
Least-squares matrix: full	Hydrogen site location: inferred from neighbouring sites
*R*[*F*^2^ > 2σ(*F*^2^)] = 0.048	H-atom parameters constrained
*wR*(*F*^2^) = 0.097	*w* = 1/[σ^2^(*F*_o_^2^) + (0.0348*P*)^2^] where *P* = (*F*_o_^2^ + 2*F*_c_^2^)/3
*S* = 1.01	(Δ/σ)_max_ < 0.001
2142 reflections	Δρ_max_ = 0.51 e Å^−^^3^
161 parameters	Δρ_min_ = −0.52 e Å^−^^3^
0 restraints	Extinction correction: *SHELXL97* (Sheldrick, 2008), Fc^*^=kFc[1+0.001xFc^2^λ^3^/sin(2θ)]^-1/4^
Primary atom site location: structure-invariant direct methods	Extinction coefficient: 0.025 (4)

### Special details

**Table:**

Geometry. All e.s.d.'s (except the e.s.d. in the dihedral angle between two l.s. planes) are estimated using the full covariance matrix. The cell e.s.d.'s are taken into account individually in the estimation of e.s.d.'s in distances, angles and torsion angles; correlations between e.s.d.'s in cell parameters are only used when they are defined by crystal symmetry. An approximate (isotropic) treatment of cell e.s.d.'s is used for estimating e.s.d.'s involving l.s. planes.
Refinement. Refinement of *F*^2^ against ALL reflections. The weighted *R*-factor *wR* and goodness of fit *S* are based on *F*^2^, conventional *R*-factors *R* are based on *F*, with *F* set to zero for negative *F*^2^. The threshold expression of *F*^2^ > σ(*F*^2^) is used only for calculating *R*-factors(gt) *etc*. and is not relevant to the choice of reflections for refinement. *R*-factors based on *F*^2^ are statistically about twice as large as those based on *F*, and *R*- factors based on ALL data will be even larger.

### Fractional atomic coordinates and isotropic or equivalent isotropic displacement parameters (Å^2^)

**Table:**

	*x*	*y*	*z*	*U*_iso_*/*U*_eq_	
Ni1	0.0000	0.5000	0.5000	0.0161 (2)	
N1	0.1088 (4)	0.7246 (3)	0.4952 (4)	0.0190 (6)	
N2	0.0726 (4)	0.7802 (4)	0.6384 (4)	0.0249 (7)	
C1	0.1491 (5)	0.9151 (5)	0.6353 (5)	0.0295 (9)	
H1	0.1204	0.9557	0.7366	0.035*	
C2	0.2687 (6)	0.9998 (5)	0.4919 (6)	0.0336 (10)	
H2	0.3228	1.0941	0.4945	0.040*	
C3	0.3045 (6)	0.9406 (5)	0.3470 (5)	0.0333 (9)	
H3	0.3853	0.9920	0.2449	0.040*	
C4	0.2185 (5)	0.8030 (4)	0.3549 (5)	0.0254 (8)	
H4	0.2391	0.7628	0.2550	0.031*	
N11	0.2622 (4)	0.4283 (3)	0.3430 (4)	0.0184 (6)	
N12	0.3917 (4)	0.4485 (4)	0.3946 (4)	0.0250 (7)	
C11	0.5593 (5)	0.4051 (5)	0.3036 (5)	0.0264 (8)	
H11	0.6503	0.4227	0.3390	0.032*	
C12	0.6091 (5)	0.3350 (5)	0.1588 (5)	0.0295 (9)	
H12	0.7298	0.3030	0.0984	0.035*	
C13	0.4777 (5)	0.3144 (4)	0.1079 (5)	0.0273 (8)	
H13	0.5035	0.2669	0.0107	0.033*	
C14	0.3019 (4)	0.3658 (4)	0.2037 (4)	0.0201 (7)	
H14	0.2092	0.3556	0.1680	0.024*	
N21	0.0470 (4)	0.4009 (4)	0.7037 (4)	0.0223 (7)	
C21	0.0896 (5)	0.3404 (4)	0.8065 (5)	0.0263 (8)	
N22	0.1310 (7)	0.2874 (5)	0.9320 (5)	0.0581 (13)	
C22	0.1948 (5)	0.1418 (5)	0.9515 (5)	0.0300 (9)	
N23	0.2467 (6)	0.0160 (5)	0.9879 (5)	0.0480 (10)	

### Atomic displacement parameters (Å^2^)

**Table:**

	*U*^11^	*U*^22^	*U*^33^	*U*^12^	*U*^13^	*U*^23^
Ni1	0.0151 (4)	0.0195 (4)	0.0144 (4)	−0.0010 (3)	−0.0066 (3)	−0.0020 (3)
N1	0.0177 (14)	0.0215 (15)	0.0193 (16)	0.0027 (12)	−0.0090 (12)	−0.0047 (12)
N2	0.0227 (16)	0.0314 (17)	0.0223 (17)	−0.0058 (13)	−0.0082 (13)	−0.0083 (13)
C1	0.032 (2)	0.029 (2)	0.033 (2)	0.0018 (17)	−0.0151 (18)	−0.0137 (17)
C2	0.037 (2)	0.0228 (19)	0.047 (3)	−0.0085 (17)	−0.021 (2)	−0.0029 (18)
C3	0.033 (2)	0.028 (2)	0.032 (2)	−0.0079 (17)	−0.0069 (18)	0.0039 (17)
C4	0.029 (2)	0.0245 (18)	0.021 (2)	−0.0027 (16)	−0.0087 (16)	0.0008 (15)
N11	0.0157 (15)	0.0224 (15)	0.0174 (16)	−0.0012 (12)	−0.0062 (12)	−0.0043 (12)
N12	0.0211 (16)	0.0290 (17)	0.0291 (18)	0.0023 (13)	−0.0131 (14)	−0.0083 (14)
C11	0.0145 (17)	0.032 (2)	0.033 (2)	−0.0030 (15)	−0.0085 (16)	−0.0050 (17)
C12	0.0207 (19)	0.030 (2)	0.031 (2)	0.0018 (16)	−0.0031 (16)	−0.0047 (17)
C13	0.0249 (19)	0.028 (2)	0.021 (2)	−0.0026 (16)	0.0003 (15)	−0.0065 (16)
C14	0.0172 (17)	0.0242 (18)	0.0162 (19)	−0.0016 (14)	−0.0036 (14)	−0.0025 (14)
N21	0.0219 (16)	0.0263 (16)	0.0191 (18)	−0.0035 (13)	−0.0079 (14)	−0.0034 (13)
C21	0.035 (2)	0.028 (2)	0.018 (2)	−0.0030 (16)	−0.0129 (17)	−0.0034 (15)
N22	0.113 (4)	0.037 (2)	0.053 (3)	0.007 (2)	−0.065 (3)	−0.0054 (19)
C22	0.037 (2)	0.035 (2)	0.024 (2)	−0.0021 (19)	−0.0190 (18)	−0.0022 (17)
N23	0.059 (3)	0.050 (2)	0.043 (2)	0.020 (2)	−0.030 (2)	−0.0134 (19)

### Geometric parameters (Å, º)

**Table:**

Ni1—N21	2.058 (3)	C4—H4	0.9500
Ni1—N21^i^	2.058 (3)	N11—C14	1.333 (4)
Ni1—N11^i^	2.125 (3)	N11—N12	1.349 (4)
Ni1—N11	2.125 (3)	N12—C11	1.330 (5)
Ni1—N1^i^	2.147 (3)	C11—C12	1.399 (5)
Ni1—N1	2.147 (3)	C11—H11	0.9500
N1—C4	1.327 (5)	C12—C13	1.359 (5)
N1—N2	1.342 (4)	C12—H12	0.9500
N2—C1	1.336 (5)	C13—C14	1.410 (5)
C1—C2	1.394 (6)	C13—H13	0.9500
C1—H1	0.9500	C14—H14	0.9500
C2—C3	1.372 (6)	N21—C21	1.148 (5)
C2—H2	0.9500	C21—N22	1.308 (5)
C3—C4	1.385 (5)	N22—C22	1.304 (6)
C3—H3	0.9500	C22—N23	1.155 (6)
			
N21—Ni1—N21^i^	180.00 (8)	C2—C3—H3	121.2
N21—Ni1—N11^i^	89.44 (11)	C4—C3—H3	121.2
N21^i^—Ni1—N11^i^	90.56 (11)	N1—C4—C3	123.2 (3)
N21—Ni1—N11	90.56 (11)	N1—C4—H4	118.4
N21^i^—Ni1—N11	89.44 (11)	C3—C4—H4	118.4
N11^i^—Ni1—N11	180.0	C14—N11—N12	120.5 (3)
N21—Ni1—N1^i^	88.23 (11)	C14—N11—Ni1	124.5 (2)
N21^i^—Ni1—N1^i^	91.77 (11)	N12—N11—Ni1	115.0 (2)
N11^i^—Ni1—N1^i^	87.48 (11)	C11—N12—N11	118.6 (3)
N11—Ni1—N1^i^	92.52 (10)	N12—C11—C12	123.7 (3)
N21—Ni1—N1	91.77 (11)	N12—C11—H11	118.1
N21^i^—Ni1—N1	88.23 (11)	C12—C11—H11	118.1
N11^i^—Ni1—N1	92.52 (10)	C13—C12—C11	117.3 (3)
N11—Ni1—N1	87.48 (11)	C13—C12—H12	121.3
N1^i^—Ni1—N1	180.0	C11—C12—H12	121.3
C4—N1—N2	120.1 (3)	C12—C13—C14	117.9 (3)
C4—N1—Ni1	121.1 (2)	C12—C13—H13	121.0
N2—N1—Ni1	118.7 (2)	C14—C13—H13	121.0
C1—N2—N1	118.4 (3)	N11—C14—C13	121.9 (3)
N2—C1—C2	123.8 (3)	N11—C14—H14	119.1
N2—C1—H1	118.1	C13—C14—H14	119.1
C2—C1—H1	118.1	C21—N21—Ni1	173.1 (3)
C3—C2—C1	116.9 (3)	N21—C21—N22	173.1 (4)
C3—C2—H2	121.6	C22—N22—C21	122.1 (4)
C1—C2—H2	121.6	N23—C22—N22	171.8 (4)
C2—C3—C4	117.6 (4)		

Symmetry code: (i) −*x*, −*y*+1, −*z*+1.

## References

[bb1] Brandenburg, K. (2011). *DIAMOND* Crystal Impact GbR, Bonn, Germany.

[bb2] Sheldrick, G. M. (2008). *Acta Cryst.* A**64**, 112–122. 10.1107/S0108767307043930 18156677

[bb3] Stoe & Cie (2008). *X-AREA*, *X-RED32* and *X-SHAPE* Stoe & Cie, Darmstadt, Germany.

[bb4] Wriedt, M. & Näther, C. (2011). *Dalton Trans.* **40**, 886–898. 10.1039/c0dt00864h 21152654

